# Using Biologic Markers in Blood to Assess Exposure to Multiple Environmental Chemicals for Inner-City Children 3–6 Years of Age

**DOI:** 10.1289/ehp.8324

**Published:** 2005-10-13

**Authors:** Ken Sexton, John L. Adgate, Ann L. Fredrickson, Andrew D. Ryan, Larry L. Needham, David L. Ashley

**Affiliations:** 1 University of Texas School of Public Health, Brownsville, Texas, USA; 2 University of Minnesota School of Public Health, Minneapolis, Minnesota, USA; 3 Centers for Disease Control and Prevention, National Center for Environmental Health, Atlanta, Georgia, USA

**Keywords:** chemical mixtures, children, cumulative exposure, environmental justice, metals, PCBs, pesticides, volatile organic compounds

## Abstract

We assessed concurrent exposure to a mixture of > 50 environmental chemicals by measuring the chemicals or their metabolites in the blood of 43 ethnically diverse children (3–6 years of age) from a socioeconomically disadvantaged neighborhood in Minneapolis. Over a 2-year period, additional samples were collected every 6–12 months from as many children as possible. We analyzed blood samples for 11 volatile organic compounds (VOCs), 2 heavy metals (lead and mercury, 11 organochlorine (OC) pesticides or related compounds, and 30 polychlorinated biphenyl (PCB) congeners. The evidence suggests that numerous VOCs originated from common sources, as did many PCBs. Longitudinal measurements indicate that between-child variance was greater than within-child variance for two VOCs (benzene, toluene), for both heavy metals (Pb, Hg), for all detectable OC pesticides, and for 15 of the measured PCB congeners (74, 99, 101, 118, 138–158, 146, 153, 156, 170, 178, 180, 187, 189, 194, 195). Despite the relatively small sample size, highest measured blood levels of 1,4-dichlorobenzene, styrene, *m*-/*p*-xylene, Pb, Hg, heptachlor epoxide, oxychlordane, dichlorodiphenyldichloroethene (*p*,*p*′-DDE), *trans*-nonachlor, and PCB congeners 74, 99, 105, 118, 138, 146, 153, 156, 170, and 180 were comparable with or higher than 95th percentile measurements of older children and adults from national surveys. Results demonstrate that cumulative exposures to multiple environmental carcinogens and neurotoxins can be comparatively high for children from a poor inner-city neighborhood.

It is well established that children are potentially at higher risk than adults for adverse health effects from exposure to many environmental chemicals (Adgate and Sexton 2001; Aprea et al. 2000; Bearer 1995; Brent and Weitzman 2004; Carlson 1998; Galson 1998; Guzelian et al. 1992; Landrigan et al. 2000; Needham and Sexton 2000). With rare exceptions, however—such as lead (National Research Council 1993a) and environmental tobacco smoke (Hecht et al. 2001; National Research Council 1986; Sexton et al. 2004a)—relatively little is known about health effects resulting from exposures to hazardous environmental chemicals for children of all ages, backgrounds, and circumstances (Adgate and Sexton 2001; Armstrong et al. 2000; Hubal et al. 2000; Needham and Sexton 2000; Selevan et al. 2000). Poor minority children may be at highest comparative risk because they tend to be both more exposed and more susceptible than the general population (Chew et al. 2003; Fox et al. 2002; Institute of Medicine 1999; Landrigan et al. 1999; Perlin et al. 2001; Sexton 1997, 1999; Sexton et al. 2004a). Nevertheless, our ability to make informed decisions about protecting their environmental health is limited by a shortage of scientific knowledge and understanding (Institute of Medicine 1999; Landrigan et al. 1998; National Research Council 1993a, 1993b; Needham and Sexton 2000; Sexton 1997; Sexton and Banks-Anderson 1993; Sexton et al. 2004b). Relatively few attempts have been made to measure children’s environmental exposure to multiple hazardous chemicals and chemical classes (Clayton et al. 2003; Sexton et al. 2003; Wilson et al. 2003). Also, the National Health and Nutrition Examination Survey (NHANES) has not assessed exposure to organochlorine (OC) pesticides, polychlorinated biphenyls (PCBs), and volatile organic compounds (VOCs) in populations < 12 years (Needham et al. 2005a). Nonetheless, there is mounting concern that related cumulative health risks may be significant [Castorina et al. 2003; Fox et al. 2002; Mileson et al. 1999; Mukerjee 1998; U.S. Environmental Protection Agency (EPA) 2003], particularly in economically disadvantaged communities (Landrigan et al. 1999; Perlin et al. 2001; Sexton 1997). In this article we summarize longitudinal biomarker measurements of exposures to VOCs, Pb, mercury, OC pesticides, and PCBs for an ethnically diverse group of children 3–6 years of age from a poor inner-city neighborhood.

## Materials and Methods

The study was conducted over a 2-year period from January 2000 through April 2002. Forty-three children from an inner-city neighborhood in Minneapolis provided at least one blood sample. Children and their mothers were recruited from those already participating in an ongoing study investigating the neuropsychological effects of Pb exposure—the Developmental Research on Attention and Memory Skills (DREAMS) study (Jordan et al. 2000). Mothers from the Phillips neighborhood were recruited into DREAMS prenatally or shortly after the birth of their child. Recruitment occurred in local clinics that serve pregnant women and offspring as well as through grassroots methods. Approval for this exposure monitoring study was obtained from the institutional review board at the University of Minnesota, and all care-givers provided verbal and written informed consent.

### Phillips neighborhood.

The Phillips neighborhood is an economically disadvantaged and ethnically diverse neighborhood of about 17,000 persons located in central Minneapolis. The median family income is $11,460, and approximately 40% of its residents live below the poverty level. Thirty-four percent of the residents are African American, 30% Native American, 22% white, 12% Southeast Asian, and 2% Hispanic or “other.” Of the 6,543 residences in Phillips, 1,950 are deemed to be “substandard structures,” and approximately 900 are condemned dwellings. The evidence indicates that Phillips is one of the most at-risk neighborhoods in Minneapolis, based on factors such as poverty, housing conditions, single-parent households, and overall morbidity and mortality (Jordan et al. 2000).

### Subjects.

Sociodemographic characteristics of children and parents participating in the study are summarized in Table 1. Almost 63% of the children were male, and 30.2% were African American, 20.9% Native American, 18.6% white, and 30.2% multiracial. Nearly 70% of the mothers were unmarried, and almost 40% had not graduated from high school, compared with 34.9% of fathers. At the time of the study, 72.1% of the mothers and 34.9% of the fathers were unemployed. In response to questions about smoking, 23.3% of the caregivers reported smoking inside the residence, and 25.6% said that there were smokers living in the household. As shown in Table 2, the child’s mean (± SD) age at the first sample collection was 2.9 ± 0.5 years, and the mother’s was 25.2 ± 6.2 years. An average of 5.1 ± 2 people lived in the child’s household.

### Data collection.

At enrollment, the child’s mother completed a baseline questionnaire that provided information about characteristics of the home environment and smoking status of occupants. Data on sociodemographic variables, such as income, education, occupation, race, and ethnicity, were obtained from the DREAMS study (Jordan et al. 2000). Families who did not have phone service were contacted by mail and asked to call the investigators. The mothers and their children came to one of two nearby clinics, where biologic samples were collected. Transportation was provided for those who needed it. Participants received $35 for each visit at which an attempt was made to collect a blood sample. The first sample was collected when the child was approximately 3 years of age, and attempts were made to collect subsequent samples every 6–12 months over a 2-year period.

### Sample handling and analysis.

Whole-blood samples were refrigerated after collection and shipped weekly (packed in freezer packs) to the National Center for Environmental Health, Centers for Disease Control and Prevention (CDC), in Atlanta, Georgia, for VOC and metals measurements. Serum was harvested from blood at the clinic, frozen, and shipped periodically to the laboratory packed in dry ice. Concentrations of 11 VOCs—1,1,1-trichloroethane, 1,4-dichlorobenzene, benzene, carbon tetrachloride, ethylbenzene, *m*-/*p*-xylene, *o*-xylene, styrene, tetrachloroethylene, toluene, trichloroethene—were measured in whole blood by gas chromatography/mass spectrometry (GC/MS) with isotope dilution quantification (a variation on the method described by Ashley et al. 1992). Levels of Pb were measured in whole blood by graphite furnace atomic absorption spectrophotometry (Miller et al. 1987), and levels of Hg were measured in whole blood using a modification of the method described by Chen et al. (1998).

Concentrations of 11 OC pesticides and related compounds and 30 PCB congeners were measured in serum by GC/high-resolution MS with isotope dilution quantification (DiPietro et al. 1997). The OC pesticides measured were dieldrin, heptachlor epoxide, hexachlorobenzene (HCB), mirex, oxychlordane, hexachlorocyclohexane (HCCH), β-HCCH, two isomeric forms of dichlorodiphenyltrichloroethane (*p*,*p*′-DDT, *o*,*p*′-DDT), a major degradate and metabolite of DDT [dichlorodiphenyl-dichloroethene (*p*,*p*′-DDE)], and *trans*-nonachlor. The PCB congeners measured were mono-*ortho*-chlorine–substituted PCBs (e.g., PCB congeners 28, 66, 74, 105, 118, 156, 157, 167), di-*ortho*-chlorine–substituted PCBs (e.g., congeners 138–158, 153, 180), and tri-*ortho*-chlorine–substituted PCBs (e.g., PCB congeners 177, 178, 183, 187).

### Statistical analysis.

We performed statistical analyses using SAS (version 9.1; SAS Institute Inc., Cary, NC) and S-Plus (MathSoft, Inc., 2000). As is common practice, we used log-transformed concentrations for all statistical tests to correct for skewness in the distributions and to normalize residuals. Where concentrations below the detection limit were not provided by the laboratory, one-half the detection limit for each chemical was used in calculations. Statistical tests included analysis of variance to calculate within-child and between-child variances, and calculation of Pearson correlation coefficients to measure associations between chemicals. Transformed means were exponentiated to obtain geometric means.

## Results

Of the 43 children who provided at least one blood sample for VOC analysis (the highest priority sample type), 42 were between 2.5 and 3.8 years and one child was 4.5 years of age. Every 6–12 months (mean ± SD, 276 ± 133 days), additional samples were collected from as many children as possible. One blood VOC sample was collected from 21 of 43 children (48.8%). Fourteen children (32.6%) provided two VOC samples, six (14.0%) provided three VOC samples, and two (4.7%) provided four VOC samples. Some children did not provide more than one sample for a variety of reasons—they had moved, changed phone numbers, did not show up for scheduled appointments, or declined further participation.

The number of valid measurements for each analyte, percentage of samples for which there was an instrument response even if it was below the limit of detection, percentage of samples above the detection limit, and concentrations of the individual compounds measured in the children’s blood are summarized in Table 3. Except for 1,1,1-trichloroethane (0.0%), carbon tetrachloride (6.3%), and trichloroethene (9.5%), we measured VOCs above their respective detection limits in > 42% of the samples analyzed (range, 42.9% for toluene to 79.4% for *m*-/*p*-xylene). Highest concentrations were measured for *m*-/*p*-xylene (median = 0.24 ng/mL, maximum = 1.4 ng/mL), 1,4-dichlorobenzene (median = 0.10 ng/mL, maximum = 27 ng/mL), and toluene (median = 0.10 ng/mL, maximum = 0.70 ng/mL).

We measured the heavy metals Pb and Hg above their detection limits in 98.3 and 51.5% of the samples, respectively. The median concentration was 2.9 μg/dL for Pb and the maximum was 21.2 μg/dL, whereas the median for Hg was 0.20 μg/L and the maximum was 5.1 μg/L.

Mirex, HCCH, and *p*,*p*′-DDT were not detected in any of the samples tested, and HCB (4.9%) and dieldrin (9.8%) were also detected infrequently. Except for *p*,*p*′-DDE, the remaining OC pesticides and related compounds were detected in between 16 and 27% of the samples tested. All serum samples contained *p*,*p*′-DDE at concentrations higher than all other OC pesticides (median = 0.30 ng/g-serum, maximum = 6.96 ng/g-serum).

Fourteen of 30 PCB congeners were not measured above their respective detection limits in any of the samples. Ten of 30 PCB congeners (66, 74, 101, 105, 110, 146, 156, 183, 187, 194) were above their respective detection limits in < 10% of the samples. The six PCB congeners measured above their respective detection limits in ≥10% of the samples were 99 (15.9%), 118 (17.1%), 138–158 (28.0%), 153 (31.7%), 170 (10.0%), and 180 (18.8%). Median concentrations for these six congeners were ≤0.03 ng/g-serum, whereas maximum values varied from 0.22 ng/g-serum for PCB-170 to 0.98 ng/g-serum for PCB-153. The median value for total PCBs, which is estimated by summing median values for PCB congeners 153, 138–158, and 180 and then multiplying by 1.54 (Needham et al. 2005b), was 0.08 ng/g-serum, whereas the maximum value, which is calculated in the same way using maximum values, was 1.75 ng/g-serum.

We examined statistical associations between pairwise combinations of measured blood concentrations for individual compounds within a particular chemical class (e.g., *m*-/*p*-xylene and ethylbenzene, Pb and Hg, oxy-chlordane and β-HCCH, PCBs 183 and 194) using Pearson correlation coefficients. For the 11 VOCs measured, *R*^2^ values were ≥0.25 for 23 of 55 pairwise combinations, and of these, *R*^2^ values were ≥0.50 for *m*-/*p*-xylene and ethylbenzene (*R* ^2^ = 0.63, *p* < 0.0001), *o*-xylene and ethylbenzene (*R* ^2^ = 0.76, *p* < 0001), and *m*-/*p*-xylene and *o*-xylene (*R*^2^ = 0.81, *p* < 0.0001). The two heavy metals, Pb and Hg, had an *R*^2^ = 0.11 (*p* = 0.19). For the eight OC pesticides and related compounds with samples above the detection limit, only 1 of 28 pairwise combinations had an *R*^2^ value > 0.16 (*R*^2^ = 0.46, *p* < 0.0001 for oxychlordane and β-HCCH). Among more than 450 pairwise combinations of PCB congeners, 64 (14%) had *R*^2^ values ≥0.50, and six (1.3%) had *R*^2^ values ≥0.75, including PCBs 180 and 187 (*R*^2^ = 0.77, *p* < 0.0001), PCBs 138–158 and 180 (*R* ^2^ = 0.80, *p* < 0.0001), PCBs 187 and 196–203 (*R* ^2^ = 0.80, *p* < 0.0001), PCBs 194 and 201 (*R*^2^ = 0.81, *p* < 0.0001), PCBs 183 and 187 (*R*^2^ = 0.83, *p* < 0.0001), and PCBs 183 and 194 (*R*^2^ = 0.87, *p* < 0.00017).

A comparison of between-child variability and within-child variability for each measured compound or group of compounds is shown in Table 4. Between-child variability was greater than within-child variability for benzene and toluene, for Pb and Hg, for all eight OC pesticides measured above their respective detection limits, and for 12 of 16 PCB congeners that were measured above their respective detection limits. Within-child variability was greater than or equal to between-child variability for six VOCs (1,4-dichlorobenzene, ethylbenzene, *m*-/*p*-xylene, *o*-xylene, styrene, and tetrachloroethylene) and for PCBs 66, 105, 110, and 183.

## Discussion

Although young children from poor neighborhoods are likely to be at higher comparative risk that the general population, we know relatively little about their exposure to environmental chemicals (Brent and Weitzman 2004; Needham and Sexton 2000; Sexton 1997). Of particular concern is the possibility that these at-risk children may suffer adverse consequences from the combined effects of exposure to multiple environmental agents (Sexton 1997; U.S. EPA 2003). However, relatively few studies have measured children’s concurrent exposure to compounds from multiple chemical classes (Adgate et al. 2000; Clayton et al. 2003; Sexton 2005; Sexton et al. 2003, 2005; Wilson et al. 2003), and only a few have looked at changes in exposure over time (Sexton 2005; Sexton et al. 2004a, 2005). Data from this study provide a novel opportunity to explore temporal changes in concurrent exposure to multiple chemicals (> 50 individual compounds) from multiple chemical classes (VOCs, heavy metals, OC pesticides, PCBs) for socioeconomically disadvantaged children.

Interpreting the data is nonetheless problematic, because exposure measurements for children 3–6 years of age are scarce, and there are few health-related benchmarks for blood levels of environmental chemicals, with Pb and carbon monoxide among the notable exceptions (Needham and Sexton 2000; Sexton et al. 2004b). To provide some context and perspective, below we compare results from the Phillips children with similar measurements in adolescents and adults who participated in NHANES. It is important to keep in mind that observed differences could be caused by differential exposures or by dissimilarities in pharmacokinetics (e.g., metabolism, excretion) and/or physiologic parameters (e.g., body weight, blood volume). For example, it is possible that children could experience exposures and uptakes similar in magnitude to nonsmoking adults, but that the levels in children’s blood would be higher because their blood volumes are smaller (e.g., ~ 2 L vs. ~ 6 L blood in an adult).

Many VOCs exhibit acute and chronic toxicity, and they are components of automotive exhaust, industrial emissions, and environmental tobacco smoke, as well as common constituents of cleaning and degreasing agents, deodorizers, dry-cleaning compounds, pesticides, personal care products, and solvents. Consequently, VOCs are ubiquitous in urban and nonurban environments, in indoor and outdoor settings, and in occupational and nonoccupational situations. The half-lives of VOCs in blood are a matter of hours, and blood levels are in the picograms per milliliter (parts per trillion) to nanograms per milliliter (parts per billion) range for most adults with no known occupational exposure. Concentrations of some VOCs, such as benzene, styrene, and toluene, are elevated in the blood of smokers (Ashley et al. 1994; Sexton et al. 2005).

Blood VOC results for children from the Phillips neighborhood and one-time measurements in a nonrepresentative sample of more than 550 adults (> 18 years of age, including smokers) with no known occupational exposure who participated in NHANES III from 1988–1994 (Ashley et al. 1994) are presented in Table 5. Blood levels of benzene (except 95th percentile values, which were substantially lower), styrene, and *m*-/*p*-xylene in the Phillips children were similar to concentrations measured as part of NHANES. On the other hand, mean, median, and 95th percentile values were noticeably higher in NHANES for 1,4-dichlorobenzene, ethylbenzene, tetrachloroethylene, toluene, 1,1,1-trichloroethane, and *o*-xylene. It is worth noting that the maximum level of 1,4-dichloro-benzene (27 ng/mL) measured in the Phillips children was three times higher than the 95th percentile NHANES value (9.2 ng/mL). Blood concentrations of carbon tetrachloride and trichloroethene were at or near the limits of detection in both studies.

Concentrations of several VOCs, particularly benzene and styrene, are known to be elevated in the blood of smokers (Ashley et al. 1994; Sexton et al. 2005). It is somewhat surprising, therefore, that blood levels of benzene and styrene in the Phillips children were comparable with concentrations measured in the NHANES sample, which included smokers. Moreover, the mean and median values for benzene and styrene in the Phillips children were higher than the values measured in 126 nonsmoking adults (benzene: mean = 0.040 ng/mL, median = < 0.030 ng/mL; styrene: mean = 0.044 ng/mL, median = 0.029 ng/mL) as part of another study (Ashley et al. 1996). Sources of VOC exposure for Phillips children were not the subject of this study, but likely sources include environmental tobacco smoke, auto exhaust, and consumer products used in the home.

In contrast to the VOCs, which are relatively short-lived in the environment (days to weeks) and in humans (hours in blood), the other compounds measured in the Phillips children are persistent chemicals that tend to endure in the environment for years and have half-lives of months to years in people (CDC 2003; Nilsson et al. 1991; Phillips et al. 1989). The primary sources of Pb exposure for children are deteriorated Pb-based paint and the related particles and chips that can contaminate dust and soil. Consumption of contaminated fish is the major source of organic Hg exposure in adults and children. Children can be exposed to OC pesticides *in utero* by means of the placenta, through breast milk, and by consuming diets that contain contaminated fats. Food is the main source of exposure to PCBs, which enter the food chain from a variety of pathways, including contaminated animal feed, accumulation in the fatty tissues of animals, and migration from packaging materials (CDC 2003; Klaassen et al. 1986).

Many of these persistent compounds, including Pb, Hg, heptachlor epoxide, oxy-chlordane, β-HCCH, *p*,*p*′-DDE, *trans*-nonachlor, and PCBs 99, 105, 118, 138, 146, 153, 156, 170, 180, and 187, are known or suspected to adversely affect neurodevelopment, and fetuses, infants, and young children are among the most susceptible (Needham et al. 2005b). Concentrations of selected neurotoxic chemicals measured in Phillips children and either children or adolescent participants in NHANES from 1999–2000 are shown in Table 6. Mean, median, and 95th percentile blood Pb concentrations were higher in the Phillips children, whereas corresponding Hg concentrations were lower. Blood levels at the 95th percentile for *p*,*p*′-DDE, *trans*-nonachlor, and PCBs 74, 99, and 138 were comparable between the two studies, whereas levels for PCBs 118, 146, 153, 156, 170, 180, and 187 were higher in adolescents participating in NHANES.

Despite the fact that < 100 samples were collected from 43 children in the Phillips neighborhood, maximum levels measured (Table 3) were comparable with or exceeded 95th percentile values from NHANES for 16 of 18 compounds listed in Table 6, including Pb, Hg, *p*,*p*′-DDE, *trans*-nonachlor, and PCBs 74, 99, 105, 118, 138, 146, 153, 156, 170, and 180. Although the sources of exposure to heavy metals, OC pesticides, and PCBs in Phillips were not investigated as part of this study, it is likely that the children were exposed to Pb from Pb-based paint chips and particles and to Hg, *p*,*p*′-DDE, *trans*-nonachlor, and PCBs as part of their diet.

Statistical associations between pairwise combinations of individual compounds suggest that many VOCs have common sources and, similarly, that many PCBs also have common sources. We found only minimal associations between Pb and Hg and between OC pesticides, suggesting that exposures were from different sources.

The ratio of between-child to within-child variability is important because it can affect determinations of the minimum sample size and number of measurements needed to detect differences between groups of individuals. Because longitudinal measurements of blood levels were made in the same children over time, it was possible to estimate within-child and between-child variability in the Phillips neighborhood. Within-child variability was greater than between-child variability for six VOCs (1,4-dichlorobenzene, ethylbenzene, *m*-/*p*-xylene, *o*-xylene, styrene, tetrachloroethylene) and PCBs 66, 105, 167, and 172, whereas between- was greater than within-child variability for benzene, toluene, Pb, Hg, all detectable OC pesticides, and PCB congeners 74, 99, 101, 118, 138–158, 146, 153, 156, 170, 178, 180, 187, 189, 194, and 195. It is not unexpected that larger within-child variability would tend to be observed for nonpersistent chemicals with shorter biologic half-lives, such as VOCs, and that larger between-child variability would tend to be observed for persistent chemicals with longer biologic half-lives, such as metals, OC pesticides, and PCBs (Needham and Sexton 2000; Needham et al. 2005a, 2005b).

Although the results of this study provide insight into the nature and magnitude of concurrent exposures for 3- to 6-year-olds living in a poor urban neighborhood, they are subject to several limitations: use of a convenience rather than a probability sample, reliance on a relatively small sample and availability of comparatively few repeat measures. Consequently, the extent to which these findings can be generalized to other populations and situations is uncertain, underscoring the need for well-designed follow-up studies.

## Conclusions

Longitudinal measurements of > 50 individual compounds in blood indicate that young children (3–6 years of age) living in a socioeconomically disadvantaged urban neighborhood were exposed concurrently to a combination of VOCs, heavy metals, OC pesticides, and PCBs. Many of these chemicals are known or suspected carcinogens and neurotoxicants, and young children may be particularly susceptible to related adverse effects. Maximum blood levels of numerous compounds equaled or exceeded 95th-percentile values measured in older children and adults from national surveys, indicating that cumulative exposures for some children may be near the high end of general population exposures. Findings demonstrate the potential of biomarkers for assessing cumulative exposure to multiple chemicals, highlight the lack of suitable comparison measurements among NHANES samples (which do not include children < 12 years of age except for Pb and Hg), and draw attention to the difficulties of determining whether differences between concentrations of blood biomarkers in children and adults (or adolescents) are related to differential exposures and/or dissimilarities in pharmacokinetics and physiologic factors.

## Figures and Tables

**Table 1:** Sociodemographic characteristics of children participating in the study (*n* = 43).

Characteristic		No.^a^ (%)
Child’s sex
Male	27 (62.8)
Female	16 (37.2)
Child’s race/ethnicity	
African American	13 (30.2)
White	8 (18.6)
Native American	9 (20.9)
Multiracial	13 (30.2)
Mother’s marital status	
Married	12 (27.9)
Unmarried	30 (69.8)
Mother’s education	
< High school	17 (39.5)
High school	13 (30.2)
Some college	7 (16.3)
College degree	4 (9.3)
Graduate degree	1 (2.3)
Father’s education	
< High school	15 (34.9)
High school	13 (30.2)
Some college	8 (18.6)
College degree	4 (9.3)
Mother’s employment	
Employed	11 (25.6)
Unemployed	31 (72.1)
Father’s employment	
Employed	27 (62.8)
Unemployed	15 (34.9)
Did caregiver ever smoke in household?	
Yes	10 (23.3)
No	31 (72.1)
Do other current smokers live in home?	
Yes	11 (25.6)
No	29 (67.4)

a The total in each category may not equal 43 because of missing values for some children.

**Table 2:** Summary of child and mother’s age at first sample collection, and number of people in the child’s household.

	Mean ± SD	Minimum–maximum
Child’s age at first sample (years)	2.9 ± 0.5	2–4
Mother’s age at first sample (years)	25.2 ± 6.2	17–40
Total no. of people in household	5.1 ± 2.0	1–10

**Table 3:** Distribution of analyte concentrations in blood for all samples obtained in the study.

						Percentile							
Analyte	No.^a^	% > 0^b^	% ≥DL	Mean ± SD	Minimum	5th	10th	25th	50th	75th	90th	95th	Maximum
VOCs (ng/mL)													
1,1,1-Trichloroethane	50	79.4	0.0	NA	NA	NA	NA	NA	NA	NA	NA	NA	NA
1,4-Dichlorobenzene	49	77.8	57.1	0.85 ± 3.85	0.00	0.04	0.04	0.05	0.10	0.31	0.98	1.80	27.00
Benzene	50	79.4	71.4	0.09 ± 0.04	0.04	0.05	0.06	0.07	0.08	0.10	0.14	0.15	0.26
Carbon tetrachloride	39	61.9	6.3	NA	NA	NA	NA	NA	NA	NA	NA	0.03	0.04
Ethylbenzene	49	77.8	74.6	0.04 ± 0.03	0.01	0.02	0.02	0.02	0.03	0.05	0.07	0.14	0.14
*m*-/*p*-Xylene	50	79.4	79.4	0.32 ± 0.24	0.09	0.10	0.11	0.13	0.24	0.44	0.64	0.72	1.40
*o*-Xylene	50	79.4	63.5	0.06 ± 0.04	0.02	0.02	0.02	0.03	0.04	0.09	0.12	0.16	0.22
Styrene	48	76.2	76.2	0.07 ± 0.03	0.02	0.03	0.04	0.05	0.07	0.07	0.09	0.11	0.18
Tetrachloroethylene	50	79.4	47.6	0.04 ± 0.04	0.02	0.02	0.02	0.02	0.03	0.05	0.11	0.13	0.16
Toluene	36	57.1	42.9	0.16 ± 0.14	0.07	0.07	0.07	0.08	0.10	0.14	0.32	0.52	0.70
Trichloroethene	50	79.4	9.5	NA	NA	NA	NA	NA	NA	NA	NA	0.01	0.02
Heavy metals
Pb (μg/dL)	58	98.3	98.3	4.46 ± 4.39	0.30	0.80	1.00	1.80	2.90	5.70	11.80	16.00	21.20
Hg (μg/L)	33	54.5	51.5	0.4 ± 0.91	0.10	0.10	0.10	0.10	0.20	0.40	1.10	1.60	5.10
OC pesticides and related compounds (ng/g-serum)													
Dieldrin	82	9.8	9.8	0.02 ± 0.01	0.01	0.01	0.01	0.01	0.01	0.01	0.02	0.04	0.08
Heptachlor epoxide	82	15.9	15.9	0.02 ± 0.02	0.01	0.01	0.01	0.01	0.01	0.01	0.03	0.04	0.12
HCB	82	4.9	4.9	0.04 ± 0.02	0.03	0.03	0.03	0.03	0.04	0.04	0.04	0.04	0.13
Mirex	81	0.0	0.0	0.01 ± 0.00	0.01	0.01	0.01	0.01	0.01	0.01	0.01	0.01	0.02
Oxychlordane	82	26.8	26.8	0.04 ± 0.06	0.01	0.01	0.01	0.01	0.01	0.05	0.11	0.15	0.30
β-HCCH	82	20.7	20.7	0.034 ± 0.05	0.01	0.01	0.01	0.01	0.01	0.01	0.07	0.11	0.29
HCCH	82	0.0	0.0	0.01 ± 0.00	0.01	0.01	0.01	0.01	0.01	0.01	0.01	0.01	0.02
*o*,*p*′-DDT	82	0.0	0.0	0.02 ± 0.00	0.02	0.02	0.02	0.02	0.02	0.03	0.03	0.03	0.03
*p*,*p*′-DDE	82	100.0	100.0	0.65 ± 0.96	0.04	0.09	0.12	0.21	0.30	0.69	1.60	2.13	6.96
*p*,*p*′-DDT	82	22.0	22.0	0.03 ± 0.02	0.02	0.02	0.02	0.02	0.03	0.03	0.07	0.08	0.11
*trans*-Nonachlor	82	24.4	24.4	0.04 ± 0.07	0.01	0.01	0.01	0.01	0.01	0.02	0.09	0.15	0.41
PCB congeners (ng/g-serum)													
PCB-66	82	42.7	2.4	0.06 ± 0.07	0.00	0.00	0.01	0.03	0.06	0.07	0.07	0.11	0.48
PCB-74	82	75.6	8.5	0.05 ± 0.06	0.00	0.00	0.00	0.01	0.04	0.05	0.09	0.20	0.34
PCB-87	82	59.8	0.0	0.01 ± 0.01	0.00	0.00	0.00	0.00	0.01	0.02	0.03	0.03	0.05
PCB-99	82	95.1	15.9	0.03 ± 0.04	0.00	0.00	0.00	0.01	0.01	0.03	0.07	0.11	0.28
PCB-101	82	64.6	2.4	0.02 ± 0.01	0.00	0.00	0.00	0.01	0.02	0.03	0.03	0.03	0.09
PCB-105	82	70.7	2.4	0.01 ± 0.01	0.00	0.00	0.00	0.00	0.01	0.02	0.03	0.03	0.06
PCB-110	82	61.0	1.2	0.02 ± 0.01	0.00	0.00	0.00	0.01	0.02	0.03	0.03	0.03	0.07
PCB-118	82	97.6	17.1	0.04 ± 0.06	0.00	0.01	0.01	0.01	0.02	0.04	0.07	0.09	0.34
PCB-128	82	35.4	0.0	0.02 ± 0.01	0.00	0.00	0.00	0.00	0.02	0.03	0.03	0.03	0.03
PCB-138–158	82	86.6	28.0	0.08 ± 0.13	0.01	0.01	0.01	0.02	0.03	0.06	0.17	0.40	0.71
PCB-146	82	59.8	4.9	0.02 ± 0.02	0.00	0.00	0.00	0.00	0.02	0.03	0.03	0.04	0.09
PCB-149	82	45.1	0.0	0.02 ± 0.01	0.00	0.00	0.00	0.00	0.02	0.03	0.03	0.03	0.03
PCB-151	82	47.6	0.0	0.01 ± 0.01	0.00	0.00	0.00	0.00	0.02	0.02	0.03	0.03	0.03
PCB-153	82	97.6	31.7	0.09 ± 0.17	0.00	0.01	0.01	0.02	0.03	0.06	0.22	0.46	0.98
PCB-156	82	62.2	6.1	0.02 ± 0.03	0.00	0.00	0.00	0.00	0.02	0.03	0.03	0.05	0.16
PCB-157	82	29.3	0.0	0.02 ± 0.01	0.00	0.00	0.00	0.02	0.02	0.03	0.03	0.03	0.04
PCB-167	82	32.9	0.0	0.02 ± 0.01	0.00	0.00	0.00	0.01	0.02	0.03	0.03	0.03	0.03
PCB-170	80	68.8	10.0	0.03 ± 0.03	0.00	0.00	0.00	0.01	0.02	0.03	0.05	0.10	0.22
PCB-172	71	36.6	0.0	0.02 ± 0.02	0.00	0.00	0.00	0.00	0.02	0.03	0.03	0.03	0.14
PCB-177	82	31.7	0.0	0.02 ± 0.01	0.00	0.00	0.00	0.01	0.02	0.03	0.03	0.03	0.03
PCB-178	82	35.4	0.0	0.02 ± 0.01	0.00	0.00	0.00	0.01	0.02	0.03	0.03	0.03	0.03
PCB-180	80	82.5	18.8	0.04 ± 0.07	0.00	0.00	0.01	0.01	0.02	0.03	0.11	0.22	0.47
PCB-183	82	45.1	1.2	0.02 ± 0.01	0.00	0.00	0.00	0.01	0.02	0.03	0.03	0.03	0.06
PCB-187	82	61.0	7.3	0.02 ± 0.02	0.00	0.00	0.00	0.01	0.02	0.03	0.04	0.06	0.10
PCB-189	81	13.6	0.0	0.02 ± 0.01	0.00	0.00	0.00	0.02	0.02	0.03	0.03	0.03	0.03
PCB-194	77	46.8	1.3	0.02 ± 0.01	0.00	0.00	0.00	0.00	0.02	0.03	0.03	0.03	0.06
PCB-195	77	26.0	0.0	0.04 ± 0.02	0.00	0.00	0.00	0.03	0.05	0.05	0.05	0.05	0.06
PCB-196–203	82	52.4	0.0	0.02 ± 0.01	0.00	0.00	0.00	0.00	0.02	0.02	0.03	0.03	0.05
PCB-206	81	43.2	0.0	0.03 ± 0.02	0.00	0.00	0.00	0.00	0.05	0.05	0.05	0.05	0.05
PCB-209	81	55.6	0.0	0.02 ± 0.02	0.00	0.00	0.00	0.00	0.00	0.05	0.05	0.05	0.05

Abbreviations: DL, detection limit; NA, not applicable (< 10% of VOC samples were greater than the detection limit).

a Includes multiple valid analytical results for those children with more than one sample.

b Percentage of samples giving an instrument response even if it was below the detection limit.

**Table 4:** Mean analyte concentrations and within-child and between-child variances among children participating in the study.

Analyte	Mean^a^ (geometric)	Between-child variance (log)	Within-child variance (log)	Ratio (between-child variance: within-child variance)
VOCs (ng/mL)				
1,1,1-Trichloroethane	NA	NA	NA	NA
1,4-Dichlorobenzene	0.16	1.79	2.05	0.87
Benzene	0.09	0.14	0.11	1.27
Carbon tetrachloride	NA	NA	NA	NA
Ethylbenzene	0.04	0.30	0.50	0.60
*m*-/*p*-Xylene	0.26	0.37	0.58	0.64
*o*-Xylene	0.05	0.31	0.59	0.53
Styrene	0.06	0.10	0.20	0.50
Tetrachloroethylene	0.04	0.40	0.44	0.91
Toluene	0.12	0.45	0.18	2.50
Trichloroethene	NA	NA	NA	NA
Heavy metals
Lead (μg/dL)	3.08	0.95	0.32	2.97
Hg (μg/L)	0.23	1.11	0.58	1.91
OC pesticides and related compounds (ng/g-serum)				
Dieldrin	0.02	0.19	0.06	3.17
Heptachlor epoxide	0.02	0.32	0.04	8.00
HCB	0.04	0.10	0.02	5.00
Mirex	0.01	0.001	0.001	1.00
Oxychlordane	0.02	1.41	0.10	14.10
β-HCCH	0.02	1.11	0.04	27.75
HCCH	0.01	0.001	0.001	1.00
*o*,*p*′-DDT	0.02	0.001	0.001	1.00
*p*,*p*′-DDE	0.37	1.89	0.10	18.90
*p*,*p*′-DDT	0.03	0.27	0.09	3.00
*trans*-Nonachlor	0.02	1.40	0.14	10.00
PCB congeners (ng/g-serum)				
PCB-66	0.04	0.90	1.23	0.73
PCB-74	0.03	2.07	1.37	1.51
PCB-87	0.01	0.94	1.02	0.92
PCB-99	0.02	1.91	0.52	3.67
PCB-101	0.01	0.89	0.72	1.24
PCB-105	0.01	1.03	1.21	0.85
PCB-110	0.01	1.15	1.17	0.98
PCB-118	0.02	1.48	0.20	7.40
PCB-128	0.01	1.00	0.98	1.02
PCB-138–158	0.04	1.83	0.08	22.88
PCB-146	0.01	1.20	0.67	1.79
PCB-149	0.01	1.24	1.17	1.06
PCB-151	0.01	1.02	0.87	1.17
PCB-153	0.04	2.61	0.14	18.64
PCB-156	0.01	1.52	1.08	1.41
PCB-157	0.02	0.98	0.86	1.14
PCB-167	0.01	1.14	1.52	0.75
PCB-170	0.01	1.72	0.69	2.49
PCB-172	0.01	1.28	1.82	0.70
PCB-177	0.01	0.93	1.05	0.89
PCB-178	0.01	1.61	1.14	1.41
PCB-180	0.02	2.28	0.37	6.16
PCB-183	0.01	0.85	0.87	0.98
PCB-187	0.02	1.15	0.43	2.67
PCB-189	0.02	1.15	0.76	1.51
PCB-194	0.01	1.33	0.70	1.90
PCB-195	0.03	1.93	0.97	1.99
PCB-196–203	0.01	1.20	1.15	1.04
PCB-206	0.01	2.39	2.15	1.11
PCB-209	0.01	3.50	3.00	1.17

NA, not applicable (< 10% of VOC samples exceeded the detection limit).

a Mean refers to the mean of means for each child with more than one sample.

**Table 5:** Comparison of blood VOC concentrations for children in the Phillips neighborhood and selected adult participants in NHANES III (ng/mL).

	Mean^a^		Median		95th Percentile	
Compound	Phillips^b^	NHANES^c^	Phillips	NHANES	Phillips	NHANES
Benzene	0.09	0.13	0.08	0.06	0.15	0.48
Carbon tetrachloride	< LOD	< LOD	< LOD	< LOD	0.03	< LOD
1,4-Dichlorobenzene	0.85	1.9	0.10	0.33	1.8^d^	9.2
Ethylbenzene	0.04	0.11	0.03	0.06	0.14	0.25
Styrene	0.07	0.07	0.07	0.04	0.11^e^	0.18
Tetrachloroethylene	0.04	0.19	0.03	0.06	0.13	0.62
Toluene	0.16	0.52	0.10	0.28	0.52	1.5
Trichloroethene	< LOD	0.02	< LOD	< LOD	0.01	0.02
1,1,1-Trichloroethane	< LOD	0.34	< LOD	0.13	< LOD	0.80
*m*-/*p*-Xylene	0.32	0.37	0.24	0.19	0.72^f^	0.78
*o*-Xylene	0.06	0.14	0.04	0.11	0.16	0.30

LOD, limit of detection.

a Arithmetic mean.

b Forty-three children from the Phillips neighborhood with at least one blood sample.

c Between 574 and 1,037 adult participants in NHANES III, depending on the VOC (Ashley et al. 1994).

d Maximum value was 27 ng/mL.

e Maximum value was 0.18 ng/mL.

f Maximum value was 1.4 ng/mL.

**Table 6:** Comparison of blood levels of chemicals associated with neurodevelopmental effects (Pb, Hg, OC pesticides, and PCB concentrations) between children in the Phillips neighborhood and children or adolescents in NHANES III.

	Geometric mean		Median		95th Percentile	
Compound	Phillips^a^	NHANES^b^	Phillips	NHANES	Phillips	NHANES
Pb (μg/dL)^c^	3.08	2.2	2.90	2.2	16.0	7.0
Hg (μg/L)^c^	0.23	0.34	0.20	0.30	1.60	2.3
Heptachlor epoxide^d^	0.02	NA	0.01	NA	0.04	NA
Oxychlordane^d^	0.02	NA	0.01	NA	0.15	NA
β-HCCH^d^	0.02	NA	0.01	NA	0.11	0.50
*p*,*p*′-DDE^d^	0.37	0.56	0.30	0.52	2.13	2.31
*trans*-Nonachlor^d^	0.02	NA	0.01	NA	0.15	0.11
PCB-74^d^	0.03	NA	0.04	NA	0.20	0.18
PCB-99^d^	0.02	NA	0.01	NA	0.11	0.11
PCB-105^d^	0.01	NA	0.01	NA	0.03	NA
PCB-118^d^	0.02	NA	0.02	NA	0.09	0.26
PCB-138^d^	0.04^e^	NA	0.03^e^	NA	0.40^e^	0.46
PCB-146^d^	0.01	NA	0.02	NA	0.04	0.08
PCB-153^d^	0.04	NA	0.03	NA	0.46	0.75
PCB-156^d^	0.01	NA	0.02	NA	0.05	0.11
PCB-170^d^	0.01	NA	0.02	NA	0.10	0.21
PCB-180^d^	0.02	NA	0.02	NA	0.22	0.54
PCB-187^d^	0.02	NA	0.02	NA	0.06	0.17

Abbreviations: < LOD, below limit of detection; NA, not applicable because geometric means were calculated only when ≥60% of samples were at or above the limit of detection, and percentile estimates were not reported below the highest limit of detection. All values in ng/g-serum, except as indicated.

a Forty-three children from the Phillips neighborhood with at least one blood sample.

b From the 1999–2000 NHANES sample (Needham et al. 2005b).

c From children 1–5 years of age in NHANES.

d From children 12–19 years of age in NHANES.

e Concentration is for PCB-138–158.
